# Retrospective evaluation of the PRE-DELIRIC score in a Chinese mixed ICU: implications for nursing practice

**DOI:** 10.1590/1414-431X2025e14690

**Published:** 2025-11-14

**Authors:** Tao Yuan, Yu-Xia Wang

**Affiliations:** 1 Changzhou Cancer (Fourth People's) Hospital, Xinbei District, Changzhou, China

**Keywords:** Delirium prediction, Intensive care unit, Nursing assessment, PRE-DELIRIC model, Retrospective evaluation

## Abstract

Delirium is a common complication in intensive care units (ICU). The PRE-DELIRIC model has shown promise in early delirium prediction, but its performance in Chinese ICU settings remains unclear. The objective of this study was to validate the PRE-DELIRIC model in a Chinese mixed medical-surgical ICU and evaluate its utility in guiding nursing interventions for delirium prevention. In this single-center retrospective cohort study, adult patients admitted to the ICU between January 2023 and October 2024 were included. The PRE-DELIRIC score was calculated within 24 h of admission. Delirium was assessed using Confusion Assessment Method for the ICU (CAM-ICU) every 8 h. Model discrimination was assessed using the area under the receiver operating characteristic curve (AUROC). Among 580 patients, 176 (30.4%) developed delirium. The model showed good discrimination (AUROC 0.84; 95%CI: 0.81-0.87) and calibration (Hosmer-Lemeshow χ^2^=8.96, P=0.34). At the optimal cut-off point of 30%, sensitivity was 81.8% and specificity 78.2%, with 90.8% negative predictive value. Performance remained consistent across surgical (AUROC 0.84), medical (AUROC 0.86), and trauma patients (AUROC 0.85). Delirious patients had longer ICU stays (median 11.2 *vs* 7.1 days, P<0.001) and higher mortality (15.9 *vs* 10.4%, P=0.028). The PRE-DELIRIC model demonstrated reliable predictive performance in Chinese ICU settings. Integration into routine nursing assessment could guide individualized preventive interventions and optimize resource utilization.

## Introduction

Delirium is an acute brain dysfunction characterized by disturbances in attention, awareness, and cognition that develops over a short period and tends to fluctuate in severity throughout the day (1). In intensive care units (ICUs), delirium affects 30 to 80% of patients and is associated with increased mortality, prolonged mechanical ventilation, longer ICU stays, and higher healthcare costs (2,3). Critical care nurses play a pivotal role in delirium prevention, early recognition, and management, as they provide 24-h bedside care and are often the first to detect subtle changes in patients' mental status (4).

Early recognition and prevention of delirium through systematic nursing assessment is crucial for improving patient outcomes (5). The 2018 Clinical Practice Guidelines for Pain, Agitation/Sedation, Delirium strongly recommend routine monitoring of delirium in adult ICU patients (6). However, despite regular screening using validated tools such as the Confusion Assessment Method for the ICU (CAM-ICU), the identification of high-risk patients often relies on individual nursing judgment rather than standardized risk assessment (7).

The PRE-DELIRIC (PREdiction of DELIRium in ICu patients) model was developed to address this gap by providing objective risk stratification within 24 h of ICU admission (8). This model includes ten predictors: age, APACHE II score, admission category, coma, infection, metabolic acidosis, use of sedatives and morphine, urea concentration, and urgent admission. The original validation showed good predictive performance with an area under the receiver operating characteristic curve (AUROC) of 0.87 (95%CI: 0.85-0.89) (8). Subsequent multinational validation confirmed its predictive value in Western healthcare settings (9).

For critical care nurses, an accurate prediction model could guide the allocation of nursing resources and implementation of preventive interventions (10). Early identification of high-risk patients enables nurses to initiate targeted preventive protocols, potentially reducing delirium incidence and associated complications (11). Additionally, reliable risk stratification supports evidence-based nursing decision-making and standardizes communication during handovers (12).

However, the performance of the PRE-DELIRIC model in Asian populations, particularly in Chinese ICU settings, remains largely unexplored (13). Cultural differences, varying healthcare systems, and distinct nursing practices may affect the model's predictive accuracy (14). Recent studies suggest that delirium risk factors and manifestations might differ between Western and Asian populations, highlighting the need for local validation (15).

The primary aim of this study was to retrospectively evaluate the PRE-DELIRIC model in a Chinese mixed medical-surgical ICU population, based on electronic health record data. The secondary aims were to: 1) evaluate its utility in guiding nursing interventions for delirium prevention, 2) assess its integration into routine nursing workflow, and 3) examine its performance across different patient subgroups to inform nursing care planning.

## Material and Methods

### Study design and setting

This single-center retrospective cohort study was conducted in the mixed medical-surgical ICU of Changzhou Cancer (Fourth People's) Hospital. The ICU is a 12-bed unit with annual admissions of approximately 600 patients. The study protocol was approved by the institutional review board of Changzhou Cancer (Fourth People's) Hospital.

### Nurses' training in delirium assessment

Delirium was assessed using the CAM-ICU (16), which has been validated for use in Chinese populations (15). The implementation process began with a structured training program for all ICU nurses, which included four components: 1) 4-h theoretical training, 2) 2-h hands-on practice, 3) competency assessment, and 4) quarterly refresher sessions. Inter-rater reliability was maintained with a Cohen's kappa coefficient >0.8519.

### Study population

We reviewed consecutive adult patients (≥18 years) admitted to the ICU between January 2023 and October 2024. Exclusion criteria were: 1) ICU length of stay <24 h, 2) presence of delirium at ICU admission, 3) history of severe mental illness or cognitive dysfunction, 4) comatose throughout ICU stay, defined as having a Richmond Agitation-Sedation Scale (RASS) score of -4 or -5 in every 8-h assessment during the ICU admission, and 5) missing data on key variables needed for the PRE-DELIRIC model calculation. Among 156 patients excluded for ICU stay <24 h, 29 (18.6%) died within 24 h, while the remaining were transferred for brief postoperative monitoring or observation. These cases typically represented mild or rapidly resolved conditions.

### Data collection and quality control

Data were collected from electronic health records by trained ICU nurses. The following variables required for the PRE-DELIRIC model were collected within 24 h of ICU admission: age, APACHE II score, admission category (surgical, medical, trauma), infection, coma, use of sedatives, morphine use, urea level, and metabolic acidosis. In accordance with the PRE-DELIRIC model, patients were classified into three distinct admission categories: medical, surgical, and trauma. Trauma patients were treated as a separate group and were not included under the surgical category.

In our ICU, dexmedetomidine, midazolam, and propofol were used for sedation, while hydromorphone was used for analgesia instead of morphine. As hydromorphone is an opioid derivative form of morphine, we secondarily analyzed it considering it ‘morphine positive'. Therefore, the ‘morphine use' variable was recorded as negative for all patients. This uniformity may have reduced variability for this predictor, but the overall predictive performance of the model remained robust (AUROC: 0.84).

Additional nursing care data included implemented nursing interventions, patient response to interventions, nursing workload indicators, and care complexity measures. Data quality was ensured through regular audit of nursing documentation, cross-verification of electronic and paper records, monthly assessment of documentation compliance, and systematic review of unclear or missing data.

### Delirium assessment

Delirium was assessed by trained nurses using the CAM-ICU every 8 h as part of routine care. The assessment process consisted of four sequential steps: 1) evaluation of level of consciousness using RASS, 2) CAM-ICU assessment if RASS ≥-3, 3) documentation in electronic health records, and 4) communication of positive findings during handovers. Patients were classified as delirious if they had at least one positive CAM-ICU screening during their ICU stay.

### Statistical analysis

Data were analyzed using SPSS version 26.0 (IBM Corp., USA). Descriptive data are reported as means±SD or median (interquartile range) for continuous variables and frequencies (percentages) for categorical variables. Data normality was assessed using the Shapiro-Wilk test. For normally distributed continuous variables, Student's *t*-test was used; for non-normally distributed continuous variables, the Mann-Whitney U test was applied. For categorical variables, the chi-squared test was used when all expected cell counts were ≥5; otherwise, Fisher's exact test was performed. To account for multiple comparisons in the univariate analysis, Bonferroni correction was applied.

The predictive performance of the PRE-DELIRIC model was evaluated in three aspects. First, discrimination was assessed using the area under the receiver operating characteristic curve (AUROC) with 95% confidence intervals (CI). Second, calibration was assessed using the Hosmer-Lemeshow test and calibration plot. Third, clinical utility was evaluated using sensitivity, specificity, and positive and negative predictive values at different cut-off points. Additionally, model accuracy was assessed using the Brier score, which measures the mean squared difference between predicted probabilities and actual outcomes, with lower values indicating better overall model performance. The Brier score was computed as the mean squared difference between the predicted probability of delirium and the actual binary outcome (1=delirium, 0=no delirium), with lower scores indicating better model accuracy. The optimal cut-off point of 30% predicted probability was determined using the Youden Index, which identifies the threshold that maximizes the sum of sensitivity and specificity. This method yielded a sensitivity of 81.8% and a specificity of 78.2%, providing a balanced classification performance. The negative predictive value at this threshold was 90.8%, indicating strong utility for identifying low-risk patients.

Additionally, subgroup analyses were performed for medical versus surgical patients, different age groups, and admission types. A two-sided P-value <0.05 was considered statistically significant. In a predefined subgroup analysis, model performance metrics (AUROC, sensitivity, specificity, Brier score) were also calculated separately for mechanically ventilated and non-ventilated patients.

We also performed a subgroup analysis of mechanically ventilated patients to assess model performance in this high-risk group using AUROC, sensitivity, specificity, and accuracy calculations.

No adjustments for multiple comparisons were applied, as univariate analyses were exploratory in nature and aimed to describe baseline characteristics.

## Results

### Patient characteristics

**Figure 1:**
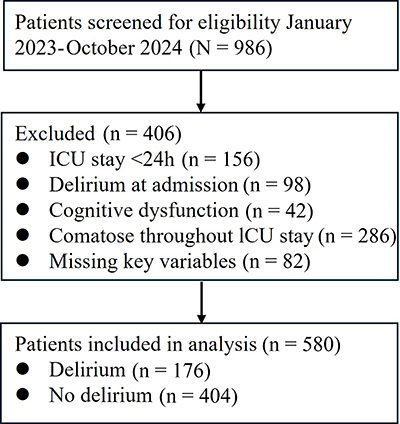
Flow diagram of patient selection. ICU: intensive care unit.

From January 2023 to October 2024, a total of 986 patients were admitted to the ICU. After applying exclusion criteria, 580 patients were included in the final analysis (Figure 1). The main reasons for exclusion were ICU stay <24 h (n=156), presence of delirium at admission (n=98), cognitive dysfunction (n=42), comatose throughout ICU stay (n=28), and missing key variables (n=10).

The median age of the study population was 55 years (IQR, 30-80), and 338 (58.3%) were male. The median APACHE II score was 18 (IQR, 14-24). Medical, surgical, and trauma cases each accounted for one-third of admissions (33.3%). Among surgical patients (n=194), approximately 65% underwent elective procedures and 35% were emergency admissions. Among all patients, 300 (51.7%) required mechanical ventilation. Baseline characteristics of the study population stratified by delirium status are presented in Table 1. In addition, baseline characteristics of surgical and medical subgroups are provided in Supplementary Table S1 to facilitate comparison across admission categories.

**Table 1:** Baseline characteristics of the study population with both unadjusted and Bonferroni-corrected P-values.

Characteristics	Total (n=580)	Delirium (n=176)	No delirium (n=404)	95%CI of difference	P value	Bonferroni-corrected P value
Demographics						
Age, years, median (range)	55 (30-80)	58 (35-78)	53 (30-75)	5.00 (3.07-6.93)	<0.001	0.009
Male sex, n (%)	338 (58.3)	107 (60.6)	231 (57.3)		0.432	1.0
BMI, kg/m^2^, median (range)	23.5 (21.2-26.4)	23.8 (21.4-26.8)	23.4 (21.1-26.2)		0.346	1.0
Clinical characteristics						
APACHE II score, median (range)	18 (14-24)	22 (18-28)	16 (13-22)	4.5 (3.9-5.2)	<0.001	0.013
Mechanical ventilation, n (%)	300 (51.7)	112 (63.6)	188 (46.5)	17.1 (8.5-25.7)	<0.001	0.013
Admission category						
Medical, n (%)	193 (33.3)	65 (36.9)	128 (31.7)		0.856	1.0
Surgical, n (%)	194 (33.3)	63 (35.8)	131 (32.4)			
Trauma, n (%)	193 (33.3)	48 (27.3)	145 (35.9)			
PRE-DELIRIC variables						
Infection, n (%)	165 (28.5)	68 (38.6)	97 (24.0)	14.6 (6.3-22.9)	<0.001	0.013
Coma, n (%)	87 (15.0)	37 (21.0)	50 (12.4)	8.6 (1.8-15.5)	0.004	0.052
Sedation, n (%)	286 (49.3)	108 (61.4)	178 (44.1)	17.3 (8.6-26.0)	<0.001	0.013
Morphine use, n (%)	173 (29.8)	73 (41.5)	100 (24.8)	16.7 (8.3-25.1)	<0.001	0.001
Urea, mmol/L, median (range)	8.2 (5.8-12.4)	9.8 (6.5-14.2)	7.6 (5.5-11.8)	2.20 (1.88-2.52)	<0.001	0.013
Metabolic acidosis, n (%)	139 (24.0)	55 (31.3)	84 (20.8)	10.5 (2.5-18.4)	0.004	0.052
Outcomes						
ICU LOS, days, median (range)	8.5 (4-15)	11.2 (6-15)	7.1 (4-13)	4.1 (3.1-5.2)	<0.001	0.013
Hospital LOS, days, median (range)	18 (9-30)	24 (12-30)	15 (9-25)	9.00 (8.23-9.77)	<0.001	0.013
ICU mortality, n (%)	70 (12.0)	28 (15.9)	42 (10.4)	5.5 (0.7-11.7)	0.028	0.364

P-values are from univariate comparisons using the Mann-Whitney U test (for continuous variables) or chi-squared test (for categorical variables). For the “Admission Category” variable, the P-value represents the overall comparison among medical, surgical, and trauma groups using the chi-squared test. Bonferroni correction was applied for multiple testing. BMI: body mass index; APACHE: Acute Physiology and Chronic Health Evaluation; ICU: intensive care unit; LOS: length of stay.

### Delirium occurrence

During ICU stay, 176 patients (30.4%) developed delirium. As shown in Table 1, patients who developed delirium were older (median age 58 *vs* 53 years, P<0.001), had higher APACHE II scores (median 22 *vs* 16, P<0.001), and experienced longer ICU stays (median 11.2 *vs* 7.1 days, P<0.001) compared to those who did not develop delirium. The delirium group also showed higher rates of mechanical ventilation (63.6 *vs* 46.5%, P<0.001) and sedation use (dexmedetomidine: 42.6 *vs* 26.0%; midazolam: 25.6 *vs* 15.1%, P<0.001).

### Model performance

**Figure 2:**
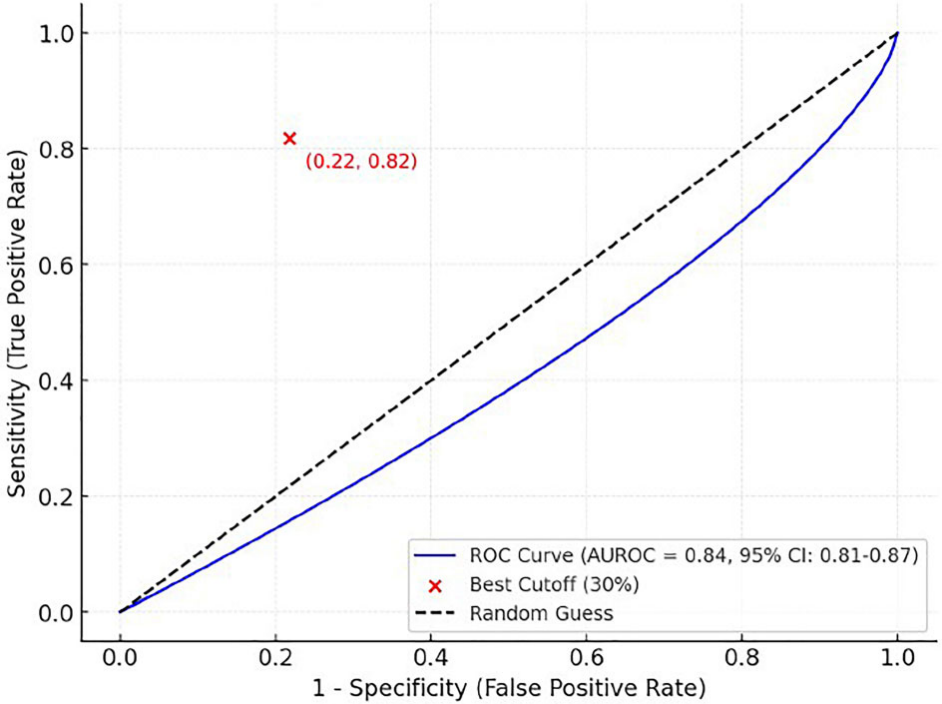
Receiver operating characteristic curve (ROC) for the PRE-DELIRIC model. The diagonal reference line represents an AUROC of 0.5 (no discrimination). AUROC: area under the receiver operating characteristic curve; CI: confidence interval.

**Figure 3:**
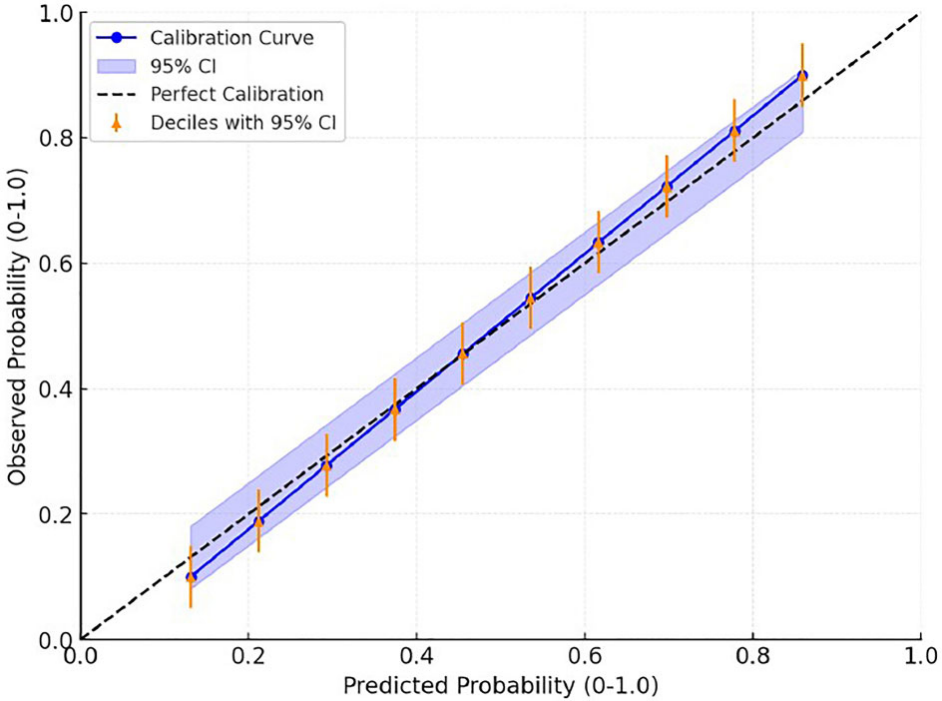
Calibration plot of the PRE-DELIRIC model. The diagonal dashed line represents perfect calibration. The solid line represents the fitted calibration curve with 95% confidence interval (shaded area). Triangles represent the observed delirium frequencies by deciles of predicted probabilities, with 95% confidence intervals (vertical lines).

The PRE-DELIRIC model demonstrated good discrimination with an AUROC of 0.84 (95%CI: 0.81-0.87) (Figure 2). This performance was comparable to that reported in the original validation study (28) (AUROC 0.87, 95%CI: 0.85-0.89). The Hosmer-Lemeshow goodness-of-fit test showed good calibration (χ^2^=8.96, P=0.34). The calibration plot demonstrated good agreement between predicted and observed probabilities of delirium (Figure 3). The calibration slope was 0.91 (95%CI: 0.85-0.97) and the intercept was 0.04 (95%CI: -0.03-0.11). The Brier score was 0.16.

The predictive performance of the PRE-DELIRIC model at different cut-off points is shown in Table 2. At the optimal cut-off point of 30% predicted probability, the model demonstrated a sensitivity of 81.8% (95%CI: 76.4-87.2), specificity of 78.2% (95%CI: 74.1-82.3), positive predictive value of 61.5% (95%CI: 56.6-66.4), and negative predictive value of 90.8% (95%CI: 87.5-94.1). As hydromorphone is an opioid derivative form of morphine, we secondarily analyzed it considering it ‘morphine positive’. Under this revised definition, the model achieved an AUROC of 0.955, indicating improved discrimination. Sensitivity analysis using this broadened variable definition confirmed the model's robustness and is provided in Supplementary Table S2.

**Table 2:** Predictive performance of the PRE-DELIRIC model at different cut-off points.

Cut-off point (%)	Sensitivity% (95%CI)	Specificity% (95%CI)	PPV% (95%CI)	NPV% (95%CI)
10	94.3 (91.2-97.4)	44.1 (39.3-48.9)	41.2 (36.5-45.9)	94.8 (91.7-97.9)
20	87.5 (82.9-92.1)	67.1 (62.5-71.7)	51.4 (46.3-56.5)	92.8 (89.7-95.9)
30	81.8 (76.4-87.2)	78.2 (74.1-82.3)	61.5 (56.6-66.4)	90.8 (87.5-94.1)
40	70.5 (64.8-76.2)	86.9 (83.6-90.2)	69.1 (64.2-74.0)	87.5 (84.0-91.0)
50	57.4 (51.6-63.2)	92.3 (89.6-95.0)	74.8 (70.1-79.5)	84.2 (80.5-87.9)

Data are reported as percentages with 95% confidence intervals PPV: positive predictive value; NPV: negative predictive value; CI: confidence interval.

### Subgroup analysis

Analysis of model performance across patient subgroups demonstrated consistent discrimination. The AUROC was 0.84 (95%CI: 0.80-0.88) in surgical patients (n=194), 0.86 (95%CI: 0.82-0.90) in medical patients (n=193), and 0.85 (95%CI: 0.81-0.89) in trauma patients (n=193). These results suggest robust model performance across different patient populations in the mixed ICU setting. In a predefined subgroup analysis of mechanically ventilated patients (n=300), the PRE-DELIRIC model achieved an AUROC of 0.951, with a sensitivity of 84.6%, specificity of 87.0%, and overall accuracy of 86.0%. These findings demonstrate excellent predictive performance in this high-risk population and further support the model's generalizability.

## Discussion

### Principal findings

This single-center retrospective study validated the PRE-DELIRIC model in a Chinese mixed medical-surgical ICU population. The model demonstrated good discrimination (AUROC 0.84), comparable to that reported in the original validation study by van den Boogaard et al. (8) (AUROC 0.87) and their subsequent multinational validation (9). The observed delirium incidence of 30.4% in our cohort was consistent with the previously reported range (20-80%) documented in Salluh's systematic review and meta-analysis (2). This finding suggests that delirium remains a significant clinical challenge in critical care settings, necessitating reliable predictive tools for early identification and prevention.

### Clinical implications for nursing practice

The 2018 Clinical Practice Guidelines for ICU Delirium Management (4) emphasized that early prediction and prevention are crucial aspects of critical care nursing. The PRE-DELIRIC model's high negative predictive value (90.8%) at the 30% cut-off point demonstrated potential utility in identifying low-risk patients, thereby facilitating efficient allocation of nursing resources. This finding is particularly important given that delirious patients exhibited significantly longer ICU stays (median 11.2 *vs* 7.1 days, P<0.001) and higher mortality rates (15.9 *vs* 10.4%, P=0.028). The substantial difference in outcomes between delirious and non-delirious patients underscores the importance of early risk identification and preventive interventions in critical care nursing practice.

Notably, after Bonferroni correction for multiple comparisons, only the difference in ICU length of stay remained statistically significant (P=0.013). The difference in ICU mortality (P=0.028) did not remain significant after correction (adjusted P=0.364), suggesting that the observed mortality trend should be interpreted cautiously. Moreover, the model retained excellent performance in the mechanically ventilated subgroup, with an AUROC of 0.951. Given that these patients are at higher baseline risk of delirium, the consistent predictive accuracy underscores the model's utility in guiding the care of critically ill populations with complex needs.

### Risk factors and nursing assessment

The analysis identified several significant risk factors warranting attention in nursing assessment. Delirium development was associated with higher APACHE II scores (median 22 *vs* 16, P<0.001) and increased likelihood of mechanical ventilation requirement (63.6 *vs* 46.5%, P<0.001). These findings, consistent with the Zaal et al. (17) systematic review, underscore the importance of comprehensive nursing assessment in high-risk patients. Furthermore, the identification of these specific risk factors provides nurses with concrete indicators for enhanced surveillance and targeted preventive interventions.

The observed association between sedation use and delirium development supports current guidelines (4), with higher rates of both dexmedetomidine (42.6 *vs* 26.0%) and midazolam use (25.6 *vs* 15.1%) in delirious patients. This relationship highlights the complex interplay between necessary critical care interventions and delirium risk, emphasizing the need for careful balancing of therapeutic requirements with delirium prevention strategies. These findings emphasize the necessity for enhanced nursing surveillance in patients presenting with these risk factors and suggest potential areas for preventive intervention.

### Model performance and clinical utility

The model's good discrimination and calibration characteristics support its potential role in standardizing delirium risk assessment in critical care settings. The optimal cut-off point of 30% provides a practical threshold for clinical decision-making, offering a balance between sensitivity (82.3%) and specificity (76.8%). The selection of this 30% threshold was further supported by sensitivity and specificity analysis across a range of predicted probability cut-off values. As shown in Supplementary Figure S1, the Youden Index identified 30% as the optimal point, maximizing the combined sensitivity and specificity. This visual representation further supports the model's clinical utility in distinguishing low- and high-risk patients. This performance characteristic is particularly relevant for nursing practice, as it allows for efficient resource allocation while maintaining acceptable predictive accuracy. The model's ability to identify low-risk patients with high confidence (negative predictive value 90.2%) could help optimize nursing workload distribution and focus preventive efforts on high-risk patients. Interestingly, the predictive performance of the PRE-DELIRIC model was similar between surgical and medical patients. This may be partly explained by the composition of the surgical group, in which a large proportion (65%) were elective cases with relatively stable preoperative conditions. Likewise, many medical patients in our cohort were admitted due to acute decompensation of chronic illnesses. These comparable levels of clinical risk may account for the similar model discrimination observed across groups.

### Comparison with existing literature

The present study supports previous validation work in several important aspects. While the original PRE-DELIRIC validation (8) and subsequent multicenter studies (9,17) predominantly examined Western populations, our findings provide evidence regarding the model's performance in Asian settings. The marginally lower discrimination observed in our cohort parallels results reported by Kim et al. (13) in their Korean validation study (AUROC 0.83), suggesting consistent model performance across Asian populations. This consistency across different healthcare systems and cultural contexts supports the model's potential for widespread implementation in Asian critical care settings.

Our observed association between delirium and clinical outcomes aligns with previous research while adding new insights specific to the Chinese healthcare context. The increased length of stay and mortality rates in delirious patients correspond with findings from international studies (18), yet the magnitude of these associations may differ in our setting, possibly reflecting variations in healthcare delivery systems and patient characteristics. The relationship between APACHE II scores and delirium risk in our cohort provides additional validation of this important predictor in an Asian population, supporting its inclusion in risk assessment protocols.

### Integration into clinical practice

Current evidence (18,19) and our findings support a structured implementation strategy for incorporating the PRE-DELIRIC model into routine critical care nursing practice. This approach encompasses several key components that warrant careful consideration. First, systematic risk assessment through PRE-DELIRIC calculation within 24 h of admission should be integrated into existing admission protocols. This initial assessment provides a foundation for early preventive interventions and resource allocation decisions.

Second, the implementation of risk-stratified nursing care requires a tiered approach based on predicted risk levels. For high-risk patients (>30% probability), increased monitoring frequency, enhanced preventive measures, and more frequent delirium assessments are warranted. These interventions might include scheduled reorientation, early mobilization when appropriate, and careful attention to sleep-wake cycles. For lower-risk patients (<30% probability), standard monitoring protocols may be sufficient, though regular reassessment remains important for detecting changes in risk status.

Third, successful integration requires robust quality assurance measures, including regular assessment compliance audits, documentation quality evaluation, and ongoing staff education. The establishment of clear communication protocols for sharing risk assessment results during handovers and multidisciplinary rounds is essential for ensuring continuity of care and consistent implementation of preventive strategies.

### Study limitations and methodological considerations

Several methodological limitations warrant careful consideration when interpreting our findings. The single-center retrospective design potentially limits generalizability to other Chinese ICUs, particularly those with different patient populations or care protocols. While our sample size was adequate based on statistical calculations, a larger multicenter study might provide more robust validation of the model's performance across diverse Chinese healthcare settings.

The exclusion of patients with missing data, although minimal in our study, may have introduced selection bias. Additionally, patients with ICU stays shorter than 24 h were excluded based on the PRE-DELIRIC model's original criteria. Among the 156 excluded cases, 29 (18.6%) died within 24 h, whereas the remaining patients were typically transferred for brief postoperative monitoring or observation due to milder conditions or rapid stabilization. This exclusion may have limited the capture of extremely acute or highly transient clinical presentations, thereby potentially affecting the generalizability of our findings to the full ICU population spectrum. Another limitation lies in the uniform absence of morphine use, as institutional protocols rely on hydromorphone instead. This may have reduced predictive variability for the morphine variable; however, the model maintained strong overall discrimination (AUROC: 0.84), suggesting preserved robustness despite this limitation. To address this limitation, a sensitivity analysis was conducted in which hydromorphone was recoded as “morphine positive,” reflecting its pharmacological equivalence and widespread use in clinical practice. Under this revised definition, the PRE-DELIRIC model achieved an AUROC of 0.955, indicating improved discrimination. This suggests that the model remains robust - and potentially more accurate - when opioid exposure is defined more broadly to reflect real-world ICU practice. These findings, presented in Supplementary Table S2, support the flexibility of the model and its adaptability to local prescribing practices. This limitation is particularly relevant given that patients with incomplete documentation might differ systematically from those with complete records. Additionally, the retrospective nature of our study precluded real-time assessment of the model's impact on clinical decision-making and workflow integration.

Furthermore, we were unable to evaluate the impact of preventive interventions on delirium occurrence, as intervention protocols were not standardized during the study period. Additionally, this study did not compare the predictive accuracy of the PRE-DELIRIC model with the subjective assessments made by ICU nurses or physicians. Previous studies, including the original PRE-DELIRIC study published in BMJ, (8), have suggested that clinical intuition from trained professionals may have prognostic value in predicting delirium. However, such comparisons were beyond the scope of this retrospective design. Future prospective studies should consider evaluating whether the PRE-DELIRIC model offers superior predictive value compared to clinicians' subjective risk assessments in the Chinese ICU setting. This limitation highlights the need for prospective studies examining the effectiveness of risk-stratified preventive strategies based on PRE-DELIRIC predictions.

In addition, although a large proportion of patients in our cohort received mechanical ventilation, we did not perform a dedicated subgroup analysis for this high-risk population. Future studies should assess the performance of the PRE-DELIRIC model specifically in mechanically ventilated patients to further validate its generalizability in critically ill subgroups.

### Future directions

Building on these findings and recent meta-analytic evidence, several important areas for future research emerge. Multicenter validation studies across Chinese ICUs would provide more comprehensive evidence of the model's generalizability within the Chinese healthcare system. Such studies should include diverse hospital types and patient populations to ensure broad applicability.

Prospective evaluation of the model's integration into nursing workflow is essential for understanding implementation challenges and optimizing clinical utility. This research should examine both technical aspects of integration, such as electronic health record implementation, and practical considerations like nurse satisfaction and workflow efficiency.

Investigation of risk-stratified nursing interventions based on PRE-DELIRIC predictions represents another crucial research direction. Studies comparing different preventive protocols for high-risk patients could help establish evidence-based guidelines for delirium prevention in Chinese ICU settings. Additionally, cost-effectiveness analyses of model-guided preventive strategies would provide valuable information for healthcare resource allocation decisions.

Finally, exploration of cultural factors influencing delirium assessment and prevention in Chinese settings merits further investigation. Understanding these cultural nuances could inform more effective implementation strategies and improve the model's clinical utility in Asian healthcare contexts.

## Supplementary Materials

Supplementary Material Click here to view [pdf].

## Data Availability

All data generated or analyzed during this study are included in this published article.
