# Supplementary material for: Retrospective evaluation of the PRE-DELIRIC score in a Chinese mixed ICU: implications for nursing practice
Source: Braz J Med Biol Res. 2025 Nov 14;58:e14690. doi: 10.1590/1414-431X2025e14690 (PMC12645441; doi:10.1590/1414-431X2025e14690)
Supplement: Supplementary file 1 [file 1414-431X-bjmbr-58-e14690-suppl.pdf]

Figure S1. Sensitivity and specificity of the PRE-DELIRIC model at varying predicted probability thresholds in the study cohort. The dashed vertical line at 30% indicates the optimal cut-off point identified via the Youden Index, balancing sensitivity (81.8%) and specificity (78.2%).

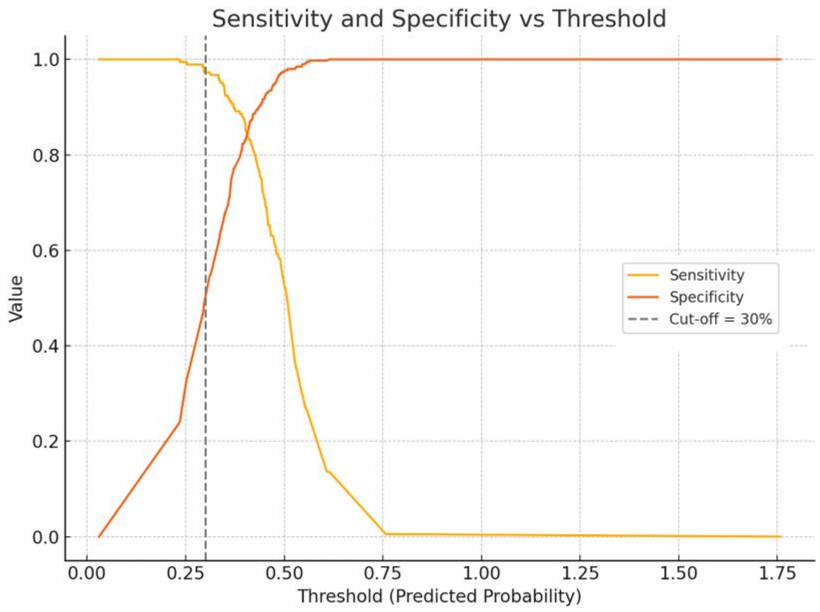

Table S1. Baseline characteristics of surgical and medical intensive care unit patients.

Variable	Surgical patients (n=194)	Medical patients (n=193)	P-value
Age, median (IQR), years	56 (42–71)	55 (38–72)	0.643
Male sex, n (%)	108 (55.7)	114 (59.1)	0.496
APACHE II score, median (IQR)	17 (14–23)	19 (15–24)	0.079
Elective surgery, n (%)	126 (65.0)	–	–
Emergency surgery, n (%)	68 (35.0)	–	–
Mechanical ventilation, n (%)	91 (46.9)	104 (53.9)	0.173
Sedative use, n (%)	71 (36.6)	82 (42.5)	0.248
Delirium occurrence, n (%)	60 (30.9)	62 (32.1)	0.804
Median ICU stay, days (IQR)	7.3 (4.9–10.8)	7.5 (5.1–11.2)	0.458
Median PRE-DELIRIC predicted risk, %	28.7 (15.6–41.2)	29.9 (16.3–-43.5)	0.523

Table S2. Sensitivity analysis of PRE-DELIRIC model performance after re-coding opioid use.

Model Definition	AUROC	Accuracy	Sensitivity	Specificity	Notes
Original (Morphine=0)	0.84	–	–	–	As per original PRE-DELIRIC specification
Expanded (Morphine + Hydromorphone)	0.955	87.4%	81.0%	90.5%	Opioid use redefined to include hydromorphone
